# The relationship between empathy and school adjustment of left-behind children: The mediating role of coping styles

**DOI:** 10.3389/fpsyg.2022.883718

**Published:** 2022-08-03

**Authors:** Guihua Qin, Ruibo Xie, Die Wang, Wei Wu, Simin Wan, Weijian Li

**Affiliations:** ^1^College of Teacher Education, Zhejiang Normal University, Jinhua, China; ^2^Research Center of Tin Ka Ping Moral Education, College of Teacher Education, Zhejiang Normal University, Jinhua, China

**Keywords:** left-behind children, empathy, school adjustment, antisocial behavior, social competence, coping styles

## Abstract

To investigate the effects of left-behind children’s empathy and coping styles on school adjustment, 605 left-behind children in the third grade from three rural elementary schools in Suzhou, Anhui Province were selected to complete the Chinese version of the Interpersonal Reaction Index Scale, the Coping Style Scale, and the School Adjustment Behavior Scale for Primary and Secondary School Students. The results showed that (1) emotional empathy positively predicted children’s social competence, and negatively predicted children’s antisocial behavior; cognitive empathy positively predicted children’s social competence; (2) the role of positive coping styles mediated the relationship between cognitive empathy and social competence, and that between cognitive empathy and antisocial behavior; negative coping styles mediated the relationship between cognitive empathy and antisocial behavior; both positive and negative coping styles mediated the relationship between emotional empathy and social competence, and that between emotional empathy and antisocial behavior. The findings of the study are valuable for understanding the relationship between empathy and school adjustment, which also helps to enhance the school adjustment of left-behind children.

## Introduction

Left-behind children are those who are left behind in rural areas under the guardianship of one parent or in the custody of grandparents or relatives because one or both parents have gone to the city to work (Fan et al., 2018). According to the annual report of the United Nations International Children’s Emergency Relief Fund in 2018, there were about 69 million left-behind children in China. Left-behind children cannot live with both parents for a long time and lack the care, support and guidance of both parents. As a result, these children are more prone to various safety and developmental problems compared to children living with both parents (Sun et al., 2015; Huang et al., 2018). For example, left-behind children reported more depression, unhealthy behaviors (Lu and Chang, 2019; Zhang et al., 2019). So we need to pay more attention to this special group. Due to the lack of family functions, when left-behind children enter the school, the school may become the main place for left-behind children to live and develop. School adjustment can reflect the development of left-behind children in the school environment, which is important for understanding the physical and mental health, and future development of left-behind children; therefore, this study will focus on examining the school adjustment of left-behind children.

Studies have examined the negative effects of parental absence on the growth and adjustment of left-behind children mainly from the perspective of their adverse situation (Wen and Lin, 2012; Wang et al., 2022). It is important to note, however, that not all left-behind children exhibit maladjustment and that left-behind children also have the potential for positive development (Hu et al., 2007). Notions of positive youth development suggests that human developmental strengths, resources, and potential are important to individual development (Damon, 2004; Kerr et al., 2010). When individuals have internal positive factors, they can still get a positive development even if in the face of adversity (Zeng and Li, 2003). This may be the main reason why some left-behind children do not exhibit school adjustment difficulties. Currently, a large body of research has shown that empathy is an important protective factor (Graf et al., 2019) and an important resource for individual environmental adaptation (Pan et al., 2012). For disadvantaged left-behind children, empathy may also be an asset that can promote positive developmental outcomes (e.g., social competence) and prevent negative outcomes (e.g., antisocial behavior). However, few studies have focused on the role of empathy in the positive development of this group. Therefore, this study will directly examine the effects of empathy on school adjustment of left-behind children and its mechanisms, which would provide insights to promote better school adjustment and healthy development for left-behind children.

### School adjustment for left-behind children

School adjustment is the individual’s ability to participate happily in school activities and to achieve academic success in a school context (Ladd et al., 1997). Children’s ability to adapt well to school learning can be reflected in both social competence and antisocial behavior (Lin et al., 2006). Social competence primarily describes adaptive or positive social behaviors, which elicits positive social outcomes. It includes interpersonal skills, self-management skills, and academic skills. Antisocial behavior primarily describes antisocial problem behaviors, which partially leads to adverse social outcomes. Hostility, aggression, and disruption are three elements of antisocial behavior (Yang et al., 2007; Li et al., 2009). Numerous studies have found that school adjustment plays a vital role in children’s academic achievement and future development. For example, research has found that children’s social competence is the basis for peer relationships and academic achievement (Malecki and Elliot, 2002; Elias and Haynes, 2008). Antisocial behavior can lead directly to various adverse outcomes, such as rejection, individual academic failure, and social and emotional discomfort in adulthood (Morison and Masten, 1991; Reijntjes et al., 2011). In addition, left-behind children are more likely to suffer from school maladjustment than children living with both parents (Hou et al., 2014). Specifically, left-behind children are less well-adjusted academically and interpersonally, are more vulnerable to peer victimization (Xu, 2010; Zhang et al., 2019), and exhibit more aggressive and disciplinary behaviors (Zhao et al., 2013). Prior studies have explored the negative effects of parental absence on school adjustment of left-behind children (Xiong et al., 2020), and the positive effects of teacher support and community support on school adjustment of left-behind children (Zhao L. L. et al., 2019). However, the disadvantageous situation of left-behind children is difficult to improve in the short term, and external factors such as community support and teacher support are uncontrollable. Therefore, protective factors within the individual left-behind children may play an active role in their development. Currently, few studies from this perspective have been conducted. Based on this, this study will explore how to promote left-behind children’s social competence, reduce antisocial behavior, and promote their healthy development based on an individual developmental perspective.

### Empathy and children’s school adjustment

Empathy refers to individual’s reaction to observing the experiences of others, an emotional response to and the understanding of the emotional and mental states of others (Davis, 1983). It includes two distinct but interrelated cognitive and affective dimensions. Cognitive empathy is the perception and understanding of another person’s internal state (Baron-Cohen and Wheelwright, 2004). Emotional empathy refers to sharing the emotions of others (Eisenberg and Miller, 1987). As an positive psychological trait in individuals (Wang and Wu, 2020), empathy is closely related to children’s social competence (Sánchez-Pérez et al., 2014) and antisocial behavior (Eisenberg et al., 2010).

Children with high empathy tend to understand the feelings of their peers and teachers, and are willing to share their emotions. In the process of interaction with others, they are easily liked by others, and often have a good teacher-student relationship and peer relationship (Boele et al., 2019; van den Bedem et al., 2019). Previous studies have shown that children’s empathy effectively promotes their learning engagement (Lee et al., 2018), and improves their social competence (Eisenberg et al., 2006). As for left-behind children, empathy is particularly essential. Supposing that these children can put themselves in their parents’ shoes, they will be more likely to understand their parents’ intentions and will have more positive feelings about their parents’ current situation away from home. All these positive thoughts and feelings will further contribute to their better communication with teachers and peers, and result in their better school adjustment. Therefore, a high level of cognitive empathy and emotional empathy is conducive to the development of left-behind children’s social competence. However, little is known about the empathy of left-behind children, and its relationship with their school adjustment has not been examined.

Besides, it is important to note that previous research indicated possible differences in the relationship between different components of empathy and children’s antisocial behavior (Moul et al., 2018). Specifically, individual emotional empathy is believed to be a crucial factor in inhibiting individual antisocial behavior (Baumeister and Lobbestael, 2011). However, studies on the effects of cognitive empathy on antisocial behavior have not yet reached a consistent conclusion. Some studies have found that cognitive empathy is not associated with outward aggression (Batanova and Loukas, 2014), nor is it significantly associated with different types of bullying (Jolliffe and Farrington, 2006). Nevertheless, some studies have shown that cognitive empathy is negatively associated with antisocial behavior (Li et al., 2015; Georgiou et al., 2019). Other studies have even suggested that children who are aggressive are more able to understand the cognition and emotions of others, and are therefore more able to manipulate others and perpetrate harm (Sutton et al., 1999a,b). That is, cognitive empathy is positively associated with bullying (Caravita et al., 2009). This inconsistency may be due to the differences in measurement instruments and study subjects. It has also been noted that cognitive empathy is an attempt to anticipate the feelings and reactions of others, which can be a trait of both a good leader and an autocrat (Kozéki and Berghammer, 1992). Therefore, the relationship between cognitive empathy and antisocial behavior still needs to be further explored. In addition, though left-behind children exhibit more antisocial behaviors (Liu et al., 2007), what exactly is the relationship between the two has not been explored by research.

In summary, this study will systematically explore the relationship between two types of empathy and two indicators of school adjustment among left-behind children. This helps to reveal the role of empathy in the positive development of the children left behind. If left-behind children possess a certain level of empathy, then they may also develop higher levels of social competence and inhibit antisocial behavior.

### The mediating role of coping styles between empathy and school adjustment

In addition to directly influencing children’s school adjustment, empathy may also have an impact on children’s school adjustment through the mediating role of coping styles. In general, coping refers to an individual’s conscious, purposeful, and flexible regulatory behavior in response to changes in the real environment, and its main function is to regulate the role of stressful events (Wang et al., 1999). The coping styles can be divided into two categories (i.e., negative coping and positive coping), which reflect emotion-centered coping and problem-centered coping, respectively (Fang et al., 2008). It has been suggested that personality traits often reflect relatively persistent, stable, and biologically based characteristics; therefore, emotional traits (such as empathy) may influence how individuals cope, which in turn may act on their behavioral outcomes (Carlo et al., 2012).

Specifically, on the one hand, empathy may influence the way individuals respond. Based on theories from empathy research, some researchers have proposed that empathy can act as a facilitator of adaptive coping, and as an inhibitor of maladaptive coping (Sun R. et al., 2019). Individuals who are more able to feel the feelings of others and recognize their thoughts and feelings usually adopt a positive coping style out of compassion and understanding for others. On the contrary, if individuals have difficulty putting themselves in the position of others, they may adopt a negative coping style based on self-centeredness. From this, empathy is a psychological resource for coping style. Some studies have shown that cognitive empathy is positively related to positive coping, and negative coping is negatively related to emotional empathy (He et al., 2020). It has also been found that individuals with stronger cognitive and emotional empathy aim primarily at resolving conflicts or easing relationships with others, and they are therefore more likely to adopt productive coping styles; in contrast, individuals with poorer cognitive and emotional empathy tend to adopt unconstructive, self-directed and ineffective coping styles (Moreno-Manso et al., 2018). For left-behind children, the lack of care and modeling from both parents may not be conducive to the development of effective coping strategies, but some studies have found that disadvantageous situations also allow some left-behind children to develop positive coping strategies (Zhao X. X. et al., 2019). We speculate that this may be related to the empathy of the left-behind children. Left-behind children with high level of empathy may receive more support and positive coping strategies in their interactions with peers and teachers, and this may compensate for the adverse effects generated by the family. Nevertheless, children with low empathy may have difficulty developing effective coping strategies, and they may adopt more negative coping styles.

On the other hand, coping styles may also affect individual’s school adjustment. When individuals are able to adopt effective coping strategies, they are usually more able to solve problems and receive more support. Thus, they are more likely to show better school adjustment. Conversely, adopting a negative coping style is bound to cause more conflicts and show poorer adjustment. Previous research has found that different forms of coping are differentially related to antisocial behavior and social competence. Problem-centered coping is associated with better psychological adjustment, whereas emotion-centered coping is associated with poorer psychological adjustment (Compas et al., 2001). Besides, more effective coping styles (e.g., problem-centered) are negatively associated with aggression, whereas less effective coping styles (e.g., emotion-centered) are positively associated with aggression (Sun P. et al., 2019). Moreover, the use of positive coping styles can facilitate individuals’ academic adjustment and reduce the manifestation of maladaptive behaviors (Sasaki and Yamasaki, 2007); positive coping styles are positively associated with academic adjustment and negative coping styles are negatively associated with academic adjustment (Quan et al., 2014). Furthermore, the coping of left-behind children is usually associated with their psychological development; specifically, negative coping styles negatively predict the mental health of left-behind children (Zhu et al., 2014), while positive coping styles may have a protective effect on the mental health of left-behind children (Jia, 2012). This may indicate that when left behind children develop positive coping styles, they are more likely to have good school adjustment.

In summary, this study hypothesized that coping styles may play mediating roles between the empathy and school adjustment. To be specific, left-behind children with a high level of empathy acquire more positive coping strategies through understanding others’ thoughts and feelings in the interactions with others, and they would show better school adjustment. On the contrary, left-behind children with a lower level of empathy have difficulty in understanding others’ thoughts and feelings, and in acquiring effective coping strategies, and they ultimately would show poorer school adjustment. Previous research has also found that coping styles might mediate the relationship between traits and behavioral outcomes (Carlo et al., 2012), which also provides a basis for the present study.

### The present study

**FIGURE 1:**
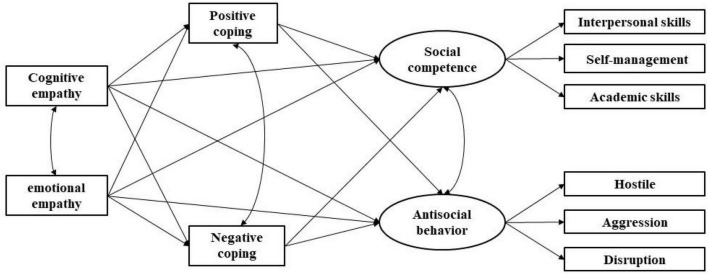
Hypothesized moderated mediation model among empathy, school adjustment, and coping styles.

Overall, empathy, as a protective factor for individuals and an important developmental asset, may play an important role in the positive development of left-behind children. This study explores the different dimensions of empathy in relation to the positive and negative aspects of school adjustment based on a developmental perspective and then explores the mediating role of coping styles. Considering that middle childhood is a critical period for individuals to develop positive psychological traits and good behaviors, and that more parents work outside the home when their children are in the lower grades (Fan, 2005), the current study focuses more on the children in the lower and middle grades. Based on the existing theoretical and empirical studies, the conceptual model is as follows (see Figure 1).

## Materials and methods

### Participants

A total of 605 left-behind children aged 9–11 years (*M*_*age*_ = 9.49, *SD*_*age*_ = 0.73; 395 boys and 210 girls) from three rural elementary schools in Anhui Province, China, participated in this study. 45 percent of children live with their mothers while their fathers works outside the home, 17 percent live with their fathers while their mothers works outside the home, and 38 percent have both parents who work outside the home.

### Procedures

The Ethics Committee of Zhejiang Normal University approved this study. All children participated voluntarily. We also acquired the consent from the schools, the teachers and the participants’ guardians. At each time point, the children completed a set of questionnaires. Two research assistants in each class explained the meaning of these items for the participants, and answered their questions. After the survey, we appreciated their participation and gave them small gifts.

### Measures

#### Interpersonal response index scale

The current study used the interpersonal response index scale developed by Davis (1980), and the Chinese version was revised by Rong et al. (2010). This scale can effectively measure empathy in elementary school children and adolescents (Mayberry and Espelage, 2007). The scale has 28 questions, all of which are scored on a 5-point scale from 1 (not at all consistent) to 5 (perfectly consistent), and includes 4 dimensions of perspective taking (e.g., “I try to look at everybody’s side of a disagreement before I make a decision.”), fantasy (e.g., “I really get involved with the feelings of the characters in a novel.”), empathic concern (e.g., “I am often quite touched by things that I see happen.”), and personal distress (e.g., “I tend to lose control during emergencies.”). Each dimension consists of 8 items. According to the current opinion of many researchers, the two dimensions of perspective selection and empathic attention can already reflect the cognitive and affective characteristics of empathy well (Hu et al., 2020; Wang and Wu, 2020). Therefore, in this study, the two dimensions of perspective taking and empathic concern were used, with perspective taking examining the degree of “understanding of others’ thoughts and feelings” and empathic concern examining the “tendency to react to others’ emotions.” The Cronbach’s alpha for this scale was 0.76.

#### Coping style scale

The “Simple Coping Style Questionnaire” developed by Xie (1998) was used to measure children’s coping styles. The 20-items questionnaire was divided into two dimensions, positive coping and negative coping. Among them, positive coping (e.g., “Don’t take the problem too seriously.”) consists of 13 items and negative coping (e.g., “Rely on others to solve problems.”) consists of 7 items. A 4-point Likert scale was used, ranging from 1 (never) to 4 (often). In the present study, the Cronbach’s alpha of this scale was 0.88.

#### School adjustment behavior scale

The School Social Behavior Scales (SSBS) Chinese revision was used to measure the school adjustment of elementary school students (Lin et al., 2006). SSBS consists of 65 items, including two dimensions of social competence and antisocial behavior. Social competence includes three factors: interpersonal skills (14 items, e.g., “Interact with many peers.”), self-management skills (10 items, e.g., “Accepted and liked by other students.”), and academic skills (8 items, e.g., “Ask the teacher questions in an appropriate manner.”). Antisocial behavior includes three factors: hostile-irritable (14 items, e.g., “Easily irritated, a little bit.”), antisocial-aggressive (10 items, e.g., “Threatening classmates and cursing.”), and demanding-destructive (9 items, e.g., “Disturbing and harassing other students.”). In the current study, the Cronbach’s alpha for this scale was 0.95.

#### Analysis

The study used SPSS 20.0 and Mplus 8.3 for data analysis. The Mean (*M*), Standard Deviation (*SD*) and Pearson’s correlations of key variables were conducted with SPSS 20.0 and Mplus 8.3 was used to verify the hypothesized model by building a structural equation modeling (SEM). Model fit was analyzed based on the ratio of chi-square (χ^2^) and the degrees of freedom, the comparative fit index (CFI), the Tucker-Lewis index (TLI), the root mean squared error of approximation (RMSEA), and the standardized root mean square residual (SRMR). Indicators of good fit were considered values of χ^2^/df of less than 7.0, values of CFI and TLI greater than 0.90, and RMSEA and SRMR lower than 0.08 (Hu and Bentler, 1999). Social competence and antisocial behavior were set as two latent variables through constructing a measurement model. The measurement model showed a good fit index [χ^2^/df = 6.874, CFI = 0.979, TLI = 0.968, SRMR = 0.029, RMSEA = 0.080 (90% CI [0.070, 0.092])], indicating that the model is acceptable for further structural model analysis. After constructing a mediating model, bootstrapping procedures were used to test indirect effects to obtain the 95% confidence intervals (CIs) of the indirect effects (Wen and Ye, 2014). Considering that child type (including father-absent, mother-absent, and both parents-absent types) and gender may influence the main variables of this study, these two variables were controlled in our analyses (Landazabal, 2009; Wu et al., 2021).

Maximum-release estimation was used to fill in the missing values. Since the study data were all self-reported from the left-behind children, a standard method bias test was conducted according to the researchers’ recommendations (Tang and Wen, 2020). A test for common method bias was performed using the Harman one-way test. The amount of variance explained by the first factor obtained with exploratory factor analysis (unrotated) was 15.04%, which is less than the critical criterion of 50% (Podsakoff and Organ, 1986), indicating that there was no significant standard method bias in this study.

## Results

### Correlations among empathy, coping styles, and school adjustment

As shown in Table 1, cognitive empathy was positively correlated with the dimensions of social competence and positive coping, and was negatively correlated with the dimensions of antisocial behavior. Emotional empathy was positively correlated with the dimensions of social competence and positive coping, was negatively correlated with the dimensions of antisocial behavior, and was not correlated with negative coping. The dimensions of social competence were positively correlated with the dimensions of coping. The dimensions of antisocial behavior were negatively correlated with positive coping, and were positively correlated with the dimensions of negative coping.

**TABLE 1:** Means, standard deviations of key variables and Pearson’s correlations between them (*N* = 605).

Variables	*M*	*SD*	1	2	3	4	5	6	7	8	9
1 Cognitive empathy	23.98	4.22	1								
2 Emotional empathy	23.60	3.88	0.477**	1							
3 Interpersonal skills	44.07	12.31	0.374**	0.261**	1						
4 Self-management	31.77	8.64	0.369**	0.236**	0.852**	1					
5 Academic skills	26.35	7.12	0.405**	0.316**	0.822**	0.794**	1				
6 Hostile	29.98	12.90	−0.171**	−0.229**	0.207**	0.207**	0.081*	1			
7 Aggression	20.05	9.82	−0.185**	−0.272**	0.166**	0.161**	0.045	0.889**	1		
8 Disruption	19.31	8.19	−0.134**	−0.188**	0.221**	0.213**	0.120**	0.877**	0.859**	1	
9 Positive coping	36.67	7.22	0.521**	0.316**	0.365**	0.353**	0.364**	−0.106**	−0.134**	–0.050	1
10 Negative coping	20.45	5.32	0.158**	−0.104*	0.164**	0.144**	0.118**	0.196**	0.217**	0.255**	0.489**

*p < 0.05, **p < 0.01.

### The mediating role of coping styles between empathy and school adjustment

**FIGURE 2:**
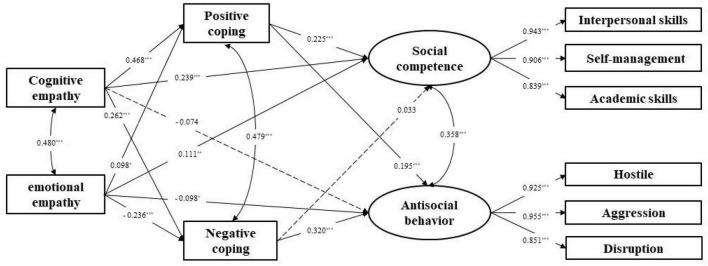
Structural equation model with standardized parameters estimates. *p < 0.05, **p < 0.01, ***p < 0.001.

After controlling for gender and child type, the prediction of empathy on social competence and antisocial behavior, and its mechanisms were examined. Cognitive empathy and emotional empathy were served as independent variables, social competence and antisocial behavior as dependent variables, and positive coping and negative coping as mediating variables. The model fit index was good [χ^2^/df = 4.290, CFI = 0.966, TLI = 0.949, SRMR = 0.037, RMSEA (90%CI) = 0.074 (0.063–0.085)]. The model results are shown in Figure 2. From the pathways in the model, cognitive empathy positively predicted social competence. Emotional empathy positively predicted social competence, and negatively predicted antisocial behavior. Cognitive empathy positively predicted positive coping style and negative coping style. Emotional empathy positively predicted positive coping style, and negatively predicted negative coping style. Positive coping negatively predicted antisocial behavior, and negative coping positively predicted antisocial behavior.

The bootstrapping method was used to test indirect effects (shown in Table 2). In this model, the direct effects of emotional empathy on antisocial behavior, emotional empathy on social competence, and cognitive empathy on social competence were all significant. The indirect effects of cognitive empathy on antisocial behavior via positive coping and negative coping were both significant. The indirect effects of cognitive empathy on social competence via positive coping was also significant. The indirect effects of emotional empathy on antisocial behavior via positive coping and negative coping were both significant. The indirect effects of emotional empathy on social competence via positive coping was also significant.

**TABLE 2:** Standardized indirect effects from empathy to school adjustment.

	Path	β	*SE*	95% CI
Total effect	Cognitive empathy → Social competence	0.354***	0.044	(0.262, 0.437)
	Cognitive empathy →Antisocial behavior	–0.085	0.041	(−0.179, 0.001)
	Emotional empathy → Social competence	0.126**	0.043	(0.042, 0.211)
	Emotional empathy → Antisocial behavior	−0.193***	0.046	(−0.282, −0.108)
Direct effect	Cognitive empathy → Social competence	0.239***	0.047	(0.144, 0.328)
	Cognitive empathy →Antisocial behavior	–0.074	0.049	(−0.172, 0.019)
	Emotional empathy → Social competenceo	0.111*	0.044	(0.023, 0.197)
	Emotional empathy → Antisocial behavior	−0.098*	0.046	(−0.187, −0.007)
Indirect effect	Cognitive empathy → Positive coping → Antisocial behavior	−0.091**	0.026	(−0.143, −0.040)
	Cognitive empathy → Negative coping → Antisocial behavior	0.084***	0.021	(0.048, 0.127)
	Emotional empathy → Positive coping → Antisocial behavior	−0.019*	0.010	(−0.040, −0.002)
	Emotional empathy → Negative coping → Antisocial behavior	−0.076***	0.017	(−0.111, −0.045)
	Cognitive empathy → Positive coping → Social competence	0.105***	0.025	(0.059, 0.156)
	Cognitive empathy → Negative coping → Social competence	0.009	0.013	(−0.017, 0.032)
	Emotional empathy → Positive coping → Social competence	0.022*	0.011	(0.002, 0.047)
	Emotional empathy → Negative coping → Social competence	–0.008	0.011	(−0.030, 0.015)

*p < 0.05, **p < 0.01, ***p < 0.001. β, standardized indirect effect; SE, Standard error; 95% CI, standardized 95% confidence intervals.

## Discussion

In this study, the effects of empathy and coping styles on school adjustment were investigated among left-behind children in the early grades. The results indicated that empathy influenced the positive development of the left behind children, and that cognitive and emotional empathy had different effects on the positive and negative aspects of school adjustment, respectively. Additionally, coping styles played a mediating role in the relationship between the above relations. These findings expand the recognition of the positive development of left-behind children. It also shows that even children in disadvantaged situations still have the potential for positive development.

### The relationship between empathy and school adjustment among left-behind children

The correlation analysis showed that both cognitive and emotional empathy were significantly and positively related to three dimensions of social competence (including interpersonal skills, self-management, and academic skills), and were negatively related to three dimensions of antisocial behavior (including hostile, aggression, and disruption). Both cognitive empathy and emotional empathy significantly and positively predicted children’s social competence, which is consistent with the results of previous studies (Boele et al., 2019). In addition, emotional empathy significantly and negatively predicted left-behind children’s antisocial behavior, which has been widely validated in existing studies on ordinary children (Baumeister and Lobbestael, 2011); whereas the relationship between cognitive empathy and antisocial behavior was not statistically significant. That is, this finding did not validate the relationship between cognitive empathy and antisocial behavior as a negative or positive predictor. These results indicate that empathy has a significant impact on left-behind children’s school adjustment, which further supports that empathy is a factor influencing individual social competence and antisocial behavior, that empathy has a significant impact on individual adjustment, development of social competence, and inhibition of aggression and disruptive behavior (Eisenberg et al., 2010; Sánchez-Pérez et al., 2014), and that there are differences in the role of cognitive empathy and emotional empathy (Vossen et al., 2015). These findings also support the idea of positive adolescent development that individuals with positive protective factors can still gain the possibility of development even in disadvantaged situations (Damon, 2004; Kerr et al., 2010).

Specifically, emotional empathy negatively predicted children’s antisocial behavior and positively predicted children’s social competence, which supports the idea that emotional empathy, as a pro-social motivator, plays a stronger role in inhibiting antisocial behavior (Lonigro et al., 2014), has a positive impact on the development of social competence, and is a powerful protective factor for left-behind children. Individuals with high level of emotional empathy typically express warmth and compassion, and concern for those who are experiencing negative experiences (Davis, 1980). Lower emotional empathy may create a greater risk of engaging in antisocial behavior (Wright et al., 2019), and hinder the development of social competence. Thus, when left-behind children are in different interpersonal situations, emotional empathy allows children to empathize with the situation and misfortune of others, which in turn inhibits behaviors such as bullying and aggression. In addition, it was found that emotional empathy was positively correlated with social competence. This finding is consistent with most existing research (Llorent García et al., 2020). When left-behind children are more able to feel the feelings of others, pay attention to their needs and act in ways that benefit others, they will also gain better interpersonal and self-management skills, and even promote the development of academic skills.

In terms of cognitive empathy, its relationship with social competence supports the results of existing studies (Boele et al., 2019). Cognitive empathy also directly and positively predicted the social competence of left-behind children. However, cognitive empathy cannot predict the antisocial behavior of left-behind children. According to Vachon et al. (2014), the ability to recognize and understand the emotions of others is a necessary component of the empathic response, but such ability does not directly lead to pro-social tendencies and is more likely to affect social competence. Cognitive empathy is an advantage in gaining dominance in certain moments (Mayberry and Espelage, 2007). For instance, in various competitive, negotiating, and aggressive relationships, high levels of cognitive empathy can be used to inflict harm and damage, and may also be used to provide assistance to others. Accordingly, the results of this study may support the idea that cognitive empathy is a neutral ability. People with high cognitive empathy may either use the emotional states of others to engage in potentially harmful behaviors or use the emotional states of others to avoid engaging in potentially harmful behaviors. Whether they exhibit antisocial behaviors may be influenced by other factors.

### The mediating role of coping styles between empathy and school adjustment

Mediating effects analysis found that coping styles mediated the association between empathy and left-behind children’s school adjustment. This result supports the claim that coping may mediate the relationship between traits and behavioral outcomes (Carlo et al., 2012). The positive prediction of emotional empathy on positive coping styles and the negative prediction of that on negative coping styles were consistent with previous studies (He et al., 2020). Emotional empathy inhibits antisocial behaviors and promotes social competence through positive coping styles. That is, left-behind children with high emotional empathy are more able to feel the thoughts and feelings of others. They adopt positive coping styles from the perspective of others and reduce negative coping styles, which in turn inhibits antisocial behaviors and facilitates the development of social competence. But, children with low emotional empathy are more likely to adopt negative coping styles, which will lead to more antisocial behaviors.

Moreover, our study found that cognitive empathy not only directly predicted social competence, but also indirectly predicted it through positive coping styles. Besides, cognitive empathy positively predicted antisocial behavior through the indirect effects of both positive and negative coping styles, which is not consistent with prior studies. He et al. (2020) found that cognitive empathy negatively predicts negative coping styles. There are multiple explanations for this inconsistent result. On the one hand, this result may further support the idea that cognitive empathy is a neutral ability, and that both positive and negative coping require a certain degree of cognitive empathy among the left-behind children. For example, individuals who use avoidance and vent emotions also need to identify the thoughts and needs of others. On the other hand, this result may also reflect the group differences of left-behind children. Parents play an essential role in developing children’s coping styles (Kliewer et al., 1996). Left-behind children without parental discipline and guidance may have more limited positive coping styles and, therefore, may adopt negative ones even if they are able to understand others. In addition, the tendency for left-behind children to be stigmatized is prominent (Zhao et al., 2016), and they may adopt more negative coping styles to conform to outside stereotypes. In addition, mediation analyses suggest that the effect of cognitive empathy on antisocial behavior may be manifested in other ways, in which positive coping reduces individual antisocial behavior and negative coping increases antisocial behavior. This is one of the more important findings of this study, namely, that cognitive empathy does not directly predict individual antisocial behavior, but can predict it through coping styles. When left-behind children adopted positive coping styles, they suppressed antisocial behavior and enhanced social competence. However, when adopting negative coping styles, their antisocial behavior would increase. This finding explains, to some extent, the mechanisms underlying the relationship between cognitive empathy and antisocial behavior and provides new evidence for understanding the relationship between cognitive empathy and antisocial behavior.

### Implications and limitations

Currently, in some countries, fostering Students’ empathy is seen as a fundamental way to address violence and bullying in schools (Lee et al., 2018). This study focuses on the association between empathy and school adjustment among left behind children, and have found that empathy is a contributing factor to the positive development of children left behind. It supports the view of positive youth development that attention should be paid to the potential expressed in the individual (Damon, 2004). Therefore external systems should provide conditions that promote the development of children’s empathy. For example, schools should focus on developing children’s empathy skills in different teaching subjects to help left-behind children better understand others’ feelings, comprehend others’ intentions, and predict others’ behaviors. In addition, left-behind children’s caregivers should focus on the coping styles of left-behind children, teaching them positive, diverse, and effective coping styles, and encouraging them to use appropriate coping styles.

However, there are some limitations in this study. First, the subjects in this study were only left-behind children around 9–11 years old, which might affect the external validity of the study. Future research may test the results of current study by investigating different age groups of left-behind children. Second, this study analyzed only the left-behind children and did not compare them with non-left-behind children, which did not help to illustrate the scope of application of the findings. In addition, there are different situations (such as children left behind living with one of the parents or grandparents), which were not explored separately in this study. Third, the variables in this study were all self-reported by children, which were highly subjective and could not exclude the influence of social approval effect. In the future, more diverse measures can be used, such as collecting assessment data from teachers and parents, or even using some methods that can obtain more objective indicators. Fourth, this study used a cross-sectional research design, and a longitudinal research design may be considered in the future to further examine the relationship between empathy and children’s school adjustment over time.

## Data availability statement

The raw data supporting the conclusions of this article will be made available by the authors, without undue reservation.

## Ethics statement

The studies involving human participants were reviewed and approved by the Zhejiang Normal University. Written informed consent to participate in this study was provided by the participants’ legal guardian/next of kin.

## Author contributions

GQ, RX, DW, WW, and WL conceived and designed the experiments. GQ and WW performed the experiments and analyzed the data. GQ drafted the manuscript. GQ, WW, DW, SW, and WL revised the manuscript. All authors read and approved the final manuscript.
